# Racial discrimination in healthcare settings and mental health among a population-based sample of racial and ethnic minoritized adults with COVID-19 in Michigan

**DOI:** 10.1016/j.pmedr.2023.102529

**Published:** 2023-11-29

**Authors:** Soomin Ryu, Jana L. Hirschtick, Kristi L. Allgood, Robert Orellana, Nancy L. Fleischer

**Affiliations:** a Department of Epidemiology, School of Public Health, University of Michigan, Ann Arbor, MI, United States of America; b Department of Epidemiology and Biostatistics, School of Public Health, Texas A&M University, College Station, TX, United States of America; c CDC Foundation, Atlanta, GA, United States of America; d Michigan Department of Health and Human Services, Lansing MI, United States of America

**Keywords:** Racial discrimination, Healthcare, Mental health, Depressive symptoms, Anxiety symptoms, COVID-19

## Abstract

The COVID-19 pandemic has worsened existing racial health disparities and racial discrimination in healthcare; however, little is known about how racial discrimination in healthcare settings is related to mental health during the pandemic. Using a population-based probability sample of racial and ethnic minoritized adults with a polymerase chain reaction (PCR)-confirmed SARS-CoV-2 infection in Michigan, we examined how measures of perceived racial discrimination in (1) seeking healthcare for COVID-19 (n = 1,210) and (2) receiving testing/treatment for COVID-19 (n = 1,364) were associated with binary variables of depressive and anxiety symptoms. We conducted a modified Poisson regression analysis with robust standard errors to estimate associations between each measure of racial discrimination and each mental health outcome separately, adjusting for demographic and socio-economic variables, health insurance, and pre-existing physical and psychiatric conditions. 7.3 % and 8.7 % of adults reported racial discrimination in seeking healthcare for COVID-19 and in getting testing/treatment for COVID–19, respectively. Although the overall prevalence of racial discrimination in healthcare settings was low, experiences of racial discrimination were associated with depressive symptoms. Adults who experienced racial discrimination in seeking healthcare had 1.74 times higher prevalence of reporting depressive symptoms (95 % CI:1.21–2.52) than those who did not. Moreover, adults who experienced racial discrimination in getting testing/treatment had 1.86 times higher prevalence of reporting depressive symptoms (95 % CI:1.36–2.53) than those who did not. Neither measure of racial discrimination was associated with anxiety symptoms in the adjusted models. There is a need for promoting anti-racial discrimination policies, educational programs, and awareness efforts in healthcare settings.

## 1 Introduction

Racial and ethnic minoritized populations have long suffered from health inequities in the U.S., with higher rates of morbidity and mortality compared to their White counterparts (Centers for Disease and Control and Prevention, 2022). These health disparities are, in large part, due to long-standing racism in the U.S., particularly in healthcare (Yearby et al., 2022). Racial discrimination in healthcare includes not only interpersonal racism, such as healthcare professionals’ unfair treatment of minoritized populations (Bird and Bogart, 2001), but also structural racism that shapes the healthcare system, resulting in inequitable access to high-quality healthcare for minoritized populations (Yearby et al., 2022). As racism is still present in the healthcare system, racial and ethnic minoritized groups have less trust in healthcare providers and system, fewer clinic visits, and lower medication adherence and medical follow-up than Whites (Stangl et al., 2019).

Growing evidence suggests that both interpersonal and structural racism are associated with worse mental and physical health (Pascoe and Richman, 2009, Williams et al., 2003). In particular, evidence consistently demonstrates that experiencing racial discrimination is closely related to poor mental health outcomes (Pascoe and Richman, 2009, Williams et al., 2003), such as depressive symptoms (Matthews et al., 2013, Woo and Jun, 2022), anxiety (Kogan et al., 2022, Sosoo et al., 2020), and poor psychological well-being (Werkuyten and Nekuee, 1999). Minority stress theory, which posits that discrimination generates a hostile and stressful social environment that affects individuals’ social and psychological functions and behaviors, explains how racial discrimination adversely affects mental health (Harrell, 2000). According to the theory, racial discrimination is a social stressor that contributes to the greater prevalence of mental health disorders among racial and ethnic minoritized groups (Wu et al., 2021). Consistent with the minority stress theory, empirical studies have documented that racial discrimination plays a significant role as a social stressor that has aggravated mental health conditions (Frost and Meyer, 2023, Pascoe and Richman, 2009, Velez et al., 2017, Williams et al., 2003).

The recent COVID-19 pandemic has exacerbated existing racial discrimination in healthcare. During the pandemic, racial and ethnic minoritized populations were more likely to be denied or lose health insurance than non-minoritized populations (CareQuest Institute for Oral Health, 2022; Gangopadhyaya et al., 2020). For example, the largest reduction in employer-sponsored health insurance was observed among Hispanic adults (-4.3 percentage points) and Asian adults (-7.6 percentage points), with small reductions among White adults (-0.8 percentage points) (Gangopadhyaya et al., 2020). Additionally, minoritized populations had limited access to COVID-19 testing, particularly at the beginning of the pandemic, due to transportation problems and fewer testing centers in their communities than White populations (Asabor et al., 2022). As a result, minoritized populations with symptoms of upper respiratory infection were less likely to be tested for COVID-19 compared to their White counterparts (Rubix Life Sciences, 2020). Moreover, the minoritized populations were also more likely to have difficulty in obtaining medication for existing chronic diseases due to poorer access to healthcare during the pandemic (Lopez et al., 2021). Many minoritized adults with COVID-19 experienced even greater discrimination and had difficulty accessing health services (Lopez et al., 2021), likely due to the intersected stigma of disease and race (Stangl et al., 2019). Racism in healthcare may contribute to racial disparities in COVID-19 treatment: the percentage of COVID-19 adult patients treated with medication was much lower among Hispanic, Black, Asian, and another race patients than among White patients (Wiltz et al., 2022).

COVID-19 has also magnified racial disparities in health outcomes, as minoritized populations have a higher likelihood of exposure to both COVID-19 and non-COVID-19 diseases due to existing structural barriers (Centers for Disease and Control and Prevention, 2021, Germain and Yong, 2020, Kim et al., 2020). For example, minoritized populations are more likely to live in crowded conditions, have jobs that cannot be performed remotely (e.g., transit workers or construction workers), and use public transportation due to lack of their own vehicle (Lopez et al., 2021). As of September 2022, American Indian, Alaska Native, Black, and Hispanic populations have experienced higher rates of hospitalizations and deaths related to COVID-19 than their White counterparts (Centers for Disease and Control and Prevention, 2021). Moreover, patients with existing medical conditions underutilize healthcare to reduce the risk of exposure to COVID-19 (CareQuest Institute for Oral Health, 2022; Kim et al., 2020), which is a more severe problem among minoritized groups due to their higher prevalence of diagnosed and undiagnosed diseases than White populations (Germain and Yong, 2020).

To date, there have been no studies that examine whether racial discrimination in healthcare settings is associated with mental health in the U.S. during the COVID-19 pandemic. Recent studies have documented that COVID-19-related racial discrimination only in general settings was associated with a higher likelihood of psychological distress, depression (Litam and Oh, 2021, Woo and Jun, 2022), and anxiety (Wu et al., 2021). Even prior to the pandemic, many studies have assessed experiences of discrimination in general settings or multiple settings, but rarely measured experiences of racism in the healthcare context (Hausmann et al., 2008, Kressin et al., 2008). Only three empirical studies before the pandemic focused on racial discrimination in healthcare to explore its relation to health outcomes in the U.S.: racial discrimination in healthcare was associated with worse glycemic control (Assari et al., 2017), poor mental health (Gee et al., 2006), and poor self-reported health (Hausmann et al., 2008).

Given that the COVID-19 pandemic has worsened existing racial discrimination in healthcare and health disparities (CareQuest Institute for Oral Health, 2022; Centers for Disease and Control and Prevention, 2021, Gangopadhyaya et al., 2020, Germain and Yong, 2020, Kim et al., 2020), there is an urgent need to understand the prevalence and influence of racial discrimination in healthcare settings related to COVID-19. Racial discrimination in healthcare may have affected mental health during the COVID-19 pandemic, which has created disease-related stigma, fear, and stress (Yuan et al., 2022). Therefore, it is timely and essential to study how experiences with racial discrimination are related to mental health outcomes in the context of COVID-19 illness. Using a population-based probability sample of racial and ethnic minoritized adults with confirmed COVID-19 in Michigan, we examined how perceived racial discrimination in seeking healthcare and receiving testing or treatment for COVID-19 was associated with depressive and anxiety symptoms. We hypothesized that experiencing racial discrimination in healthcare settings for COVID-19 care would be associated with greater depressive and anxiety symptoms. Our findings will provide new information on the prevalence and influence of understudied racial discrimination in the healthcare setting, particularly in the context of a public health emergency, which has crucial public health and policy implications.

## 2 Methods

### 2.1 Sample

We used data from the Michigan COVID-19 Recovery Surveillance Study (MI CReSS), a statewide representative survey of adults 18 years and older with a polymerase chain reaction (PCR)–confirmed SARS-CoV-2 test in the Michigan Disease Surveillance System (MDSS). A stratified probability sample of eligible adults was selected from geographic strata, including six public health emergency preparedness regions (Michigan Department of Health and Human Services, 2020), six counties (Macomb, St. Clair, Washtenaw, Oakland, Monroe, Wayne, excluding Detroit), and one city (Detroit). We included adults with COVID-19 onset during January 1, 2020–May 31, 2022. Respondents completed surveys between June 2020 and December 2022. Samples were drawn over time with a base number of 50–70 individuals from each geographic region, while the remainder of the sample was drawn proportionally based on overall case counts within each area. Non-institutionalized adults were eligible for the sampling frame if they were alive at the time the survey was conducted and had a valid phone number and zip code or county information. Respondents completed the questionnaire either online in English or over the phone with an interviewer in English, Spanish, or Arabic. The weights matched the age and sex distribution of the sampling frame in each geographic area. The median time from illness onset to survey completion was 4.4 months (IQR = 3.4–5.7 months) and the response rate was 32.1 % (American Association for Public Opinion Research, 2016).

The current analysis excluded non-Hispanic White respondents and respondents with missing information on the exposure, outcome, or covariates, except for missing household income information, which was imputed using the weighted sequential hot-deck method and hot deck propensity score imputation. Of the 5,528 total respondents in the sixteen MI CReSS waves, non-Hispanic White respondents (n = 3,852) and respondents with missing outcome or covariate information (n = 292) were excluded. An additional 3 surveys collected via proxy respondents due to mental capacity issues were also excluded from the analysis. We have separate analytic samples depending on the independent variable due to a different number of missing records. The first analytic sample for seeking healthcare for COVID-19 was n = 1,210, and the second sample for getting testing or treatment for COVID-19 was n = 1,364. The University of Michigan institutional review board deemed this study exempt.

## 3 Dependent variables

Our dependent variables were depressive and anxiety symptoms. We assessed depressive symptoms based on responses to the Patient Health Questionnaire two-item survey, which asks respondents over the last two weeks, “How often have you been bothered by having little interest or pleasure in doing things?” and “How often have you been bothered by feeling down, depressed or hopeless?” (Kroenke et al., 2003). Presence of current anxiety symptoms was assessed using the two-item Generalized Anxiety Disorder survey, which asked respondents over the last two weeks, “How often have you been bothered by feeling nervous, anxious or on edge?” and “How often have you been bothered by not being able to stop or control worrying?” (Sapra et al., 2020). Each item was rated on a 4-point Likert scale, with options including “never (0),” “for several days (1),” “for more than half the days (2),” and “nearly every day (3).” Ratings were summed within each 2-item construct. Using a cut-off score of 3+ (Kroenke et al., 2003), we created two binary variables indicating the presence of depressive symptoms or anxiety symptoms.

## 4 Independent variables

Our two independent variables were perceived racial discrimination in (1) seeking healthcare for COVID-19 and (2) getting testing or treatment for COVID-19. Both measures were adapted from the Behavioral Risk Factor Surveillance System survey (MacIntosh et al., 2013). Racial discrimination in seeking healthcare for COVID-19 was assessed using the question, “When you were seeking healthcare for COVID-19, did you feel your experiences were worse than, the same as, or better than people of other races?” Responses options included “worse than other races,” “the same as other races,” “better than other races,” “worse than some races, better than others,” “only encountered people of the same race,” and “no healthcare for COVID-19.” Based on previous literature, we categorized perceived discrimination in seeking healthcare into a binary variable: 1 for responses “worse than other races” or “worse than some, better than others” and 0 for responses “the same as other races,” or “better than other races.” (Grandner et al., 2012).

Racial discrimination in getting testing or treatment for COVID-19 was evaluated using the question, “Thinking about testing or treatment for COVID-19, did you ever feel emotionally upset, for example, angry, sad, or frustrated, as a result of how you were treated based on your race?” We coded perceived discrimination in getting testing or treatment for COVID-19 as a binary variable (1= “yes”; 0= “no”) based on prior literature (Stone and Carlisle, 2019).

## 5 Covariates

We included several demographic, socioeconomic, and health status characteristics: age (18–34, 35–44, 45–54, ≥55), sex (male, female), race and ethnicity (Hispanic, non-Hispanic Black, another non-Hispanic race and ethnicity), marital status (married/cohabiting, not currently married/cohabiting), education (≤high school graduates, some college, college graduates), household income (<$35,000, $35,000–74,999, ≥$75,000), employment status (employed, unemployed [out of work, homemaker, student, retired, unable to work]), health insurance (private insurance, Medicare/Medicaid/another type, none), pre-existing diagnosed physical comorbidities (presence of asthma, diabetes, high blood pressure, chronic obstructive pulmonary disease, cardiovascular disease, liver disease, kidney disease, cerebrovascular disease, cancer, immunosuppressive condition, autoimmune condition, or physical disability), and pre-existing diagnosed psychological or psychiatric condition. Additionally, we adjusted for survey type (phone, online) and year of COVID-19 infection (2020, 2021, 2022).

### 5.1 Statistical analysis

We first calculated weighted prevalence estimates of the two measures of racial discrimination in a healthcare setting overall and by sociodemographic and health status covariates. Then, we conducted unadjusted and adjusted Poisson regression models with robust standard errors to estimate associations between the two measures of racial discrimination in a healthcare setting and depressive or anxiety symptoms, separately. Adjusted regression models included all covariates. We conducted statistical analyses using Stata, version 17 and accounted for the complex survey design.

## 6 Results

Table 1 presents the population distribution and prevalence of perceived racial discrimination in healthcare by sociodemographic and health status factors. Nearly 18 % of racial and ethnic minoritized adults had depressive symptoms and approximately 21 % had anxiety symptoms. The prevalence of racial discrimination in seeking healthcare for COVID-19 was 7.3 %, and the prevalence of racial discrimination in getting testing or treatment for COVID-19 was 8.7 %. Both measures were more prevalent among adults who had depressive symptoms (12.5 % and 15.0 %) than those who did not have depressive symptoms (6.2 % and 7.2 %). Racial discrimination in seeking healthcare was more prevalent among adults who were non-Hispanic Black (11.9 %) than those who were Hispanic (4.7 %) or another non-Hispanic race and ethnicity (5.1 %), while racial discrimination in getting testing or treatment was prevalent among adults who were not married or cohabiting (10.9 %) than adults who were either married or cohabiting (6.2 %). Both racial discrimination measures were more prevalent among adults who had pre-existing physical comorbidities (9.1 % and 11.2 %) than adults who did not have (5.5 % and 5.9 %).

**Table 1:** Population distribution and prevalence of racial discrimination in healthcare among racial and ethnic minoritized adults by sociodemographic and clinical factors, Michigan COVID-19 Recovery Surveillance Study, 2020–2022.

	Racial discrimination in seeking healthcare for COVID-19 (n = 1,210)					Racial discrimination in getting testing/treatment for COVID-19 (n = 1,364)			
	Population distribution	Prevalence				Population distribution	Prevalence		
			95 % CI					95 % CI	
		Weighted row %	LB	UB			Weighted row %	UB	UB
Total sample		7.3	5.9	9.1			8.7	7.2	10.5
Depressive symptoms									
Yes	17.9	12.5	8.5	18.0		17.9	15.0	10.8	20.5
No	82.1	6.2	4.9	8.0		82.1	7.2	5.8	9.0
Anxiety symptoms									
Yes	21.3	10.6	6.8	14.8		21.1	10.6	7.4	14.8
No	78.7	6.6	5.1	8.4		78.9	8.1	6.6	10.0
Age group									
18–34	42.4	6.7	4.7	9.6		42.2	6.3	4.3	9.0
35–44	19.7	6.7	4.1	10.8		19.8	9.7	6.5	14.1
45–54	17.6	9.2	6.0	13.8		17.3	12.5	8.9	17.3
≥55	20.2	7.7	5.0	11.7		20.8	9.2	6.5	13.0
Sex									
Female	54.7	8.5	6.6	10.8		54.3	7.8	5.8	10.6
Male	45.3	6.0	4.1	8.7		45.7	9.3	7.4	11.6
Race and ethnicity									
Hispanic	25.0	4.7	2.7	7.9		25.5	6.9	4.6	10.4
Non-Hispanic Black	35.4	11.9	9.1	15.4		35.9	9.9	7.6	12.9
Another non-Hispanic race and ethnicity	40.0	5.1	3.3	7.5		38.6	8.6	6.3	11.6
Marital status									
Married/cohabiting	53.0	7.4	5.4	9.9		52.6	6.2	4.5	8.3
Not married/cohabiting	47.0	7.3	5.5	9.8		47.4	10.9	8.7	13.5
Education									
≤High school graduates	34.8	6.3	4.3	9.2		35.2	8.9	6.6	12.0
Some college	32.6	9.1	6.6	12.5		32.1	9.5	7.0	12.7
College graduates	32.6	6.7	4.5	9.8		32.7	7.5	5.2	10.5
Household income									
<$35,000	44.8	8.8	6.6	11.6		45.9	8.8	6.8	11.3
$35,000–74,999	28.9	6.9	4.6	10.2		28.2	9.7	7.0	13.3
≥$75,000	26.3	5.4	3.3	8.8		26.0	7.2	4.8	10.8
Employment status									
Employed	70.2	7.5	5.9	9.6		69.6	8.3	6.6	10.3
Unemployed	29.9	7.0	4.7	10.3		30.4	9.4	6.9	12.7
Health insurance status									
No health insurance	13.7	10.0	5.9	16.5		13.2	12.2	7.7	18.6
Private health insurance	54.8	6.1	4.5	8.2		55.0	7.3	5.6	9.4
Medicare/Medicaid/others	31.5	8.4	5.9	11.8		31.8	9.5	7.0	12.8
Pre-existing physical diagnosed comorbidities									
Yes	50.7	9.1	7.0	11.8		51.0	11.2	9.0	13.9
No	49.3	5.5	3.9	7.8		49.0	5.9	4.3	8.2
Pre-existing psychological, psychiatric condition									
Yes	10.7	8.4	4.7	14.7		11.1	9.3	5.5	15.2
No	89.3	7.2	5.8	9.0		88.9	8.6	7.0	10.4
**Notes**: COVID-19 = coronavirus disease 2019; CI = confidence interval; LB = lower bound; UB = upper bound; Weighted percentages are reported.									

Table 2 reports the results of Poisson regression models to examine the associations between racial discrimination in seeking healthcare for COVID-19 and depressive or anxiety symptoms. Prevalence ratios (PR) and 95 % confidence intervals (CI) are reported. In the unadjusted model, racial and ethnic minoritized adults who experienced racial discrimination in seeking healthcare for COVID-19 had 1.80 times higher prevalence of depressive symptoms (95 % CI: 1.25–2.58). After adjusting for all covariates, the association was slightly attenuated but remained statistically significant (aPR: 1.74, 95 % CI: 1.21–2.52). On the contrary, racial discrimination in seeking healthcare for COVID-19 was not associated with the prevalence of anxiety symptoms (PR: 1.42, 95 % CI: 0.99–2.05; aPR: 1.32, 95 % CI: 0.92–1.89).

**Table 2:** Poisson regression: Racial discrimination in seeking healthcare for COVID-19, depressive symptoms, and anxiety symptoms among racial and ethnic minoritized adults, Michigan COVID-19 Recovery Surveillance Study, 2020–2022 (n = 1,210).

**Dependent variable:**	**Have depressive symptoms** **(yes/no)**		**Have anxiety symptoms** **(yes/no)**	
	Unadjusted PR (95 % CI)	Adjusted PR (95 % CI)	Unadjusted PR (95 % CI)	Adjusted PR (95 % CI)
Experienced racial discrimination in seeking healthcare	1.80**	1.74**	1.42†	1.32
	(1.25–2.58)	(1.21–2.52)	(0.99–2.05)	(0.92–1.89)
Age group (ref: ≥55)				
18–34		1.12		1.99***
		(0.78–1.62)		(1.37–2.89)
35–44		0.97		1.39
		(0.64–1.46)		(0.91–2.12)
45–54		1.16		1.35
		(0.79–1.71)		(0.90–2.02)
Sex (ref: male)				
Female		1.00		1.46**
		(0.77–1.30)		(1.14–1.87)
Race and ethnicity (ref: Hispanic)				
Non-Hispanic Black		0.90		0.98
		(0.66–1.24)		(0.73–1.31)
Another non-Hispanic race/ethnicity		0.71*		0.87
		(0.51–0.98)		(0.65–1.15)
Marital status (ref: not married/cohabiting)				
Married/cohabiting		0.72*		0.86
		(0.55–0.94)		(0.68–1.08)
Education (ref: college graduates)				
≤High school graduates		1.49*		1.60**
		(1.03–2.16)		(1.13–2.25)
Some college		1.14		1.42*
		(0.79–1.64)		(1.02–1.97)
Household income (ref: ≥$75,000)				
<$35,000		1.06		1.38
		(0.70–1.61)		(0.93–2.04)
$35,000–74,999		1.07		1.28
		(0.71–1.62)		(0.87–1.88)
Employment status (ref: unemployed)				
Employed		0.94		1.04
		(0.72–1.23)		(0.82–1.32)
Health insurance status (ref: private insurance)				
No health insurance		1.40†		0.88
		(0.98–2.00)		(0.62–1.25)
Medicare/Medicaid/others		1.09		1.02
		(0.80–1.48)		(0.78–1.34)
Pre-existing physical comorbidities		1.35*		1.45**
		(1.01–1.78)		(1.14–1.85)
Pre-existing psychological/psychiatric condition		3.27***		2.62***
		(2.53–4.22)		(2.07–3.31)

**Notes:** COVID-19 = coronavirus disease 2019; PR = prevalence ratio; CI = confidence interval; The adjusted model included survey mode and year of COVID-19 infection

†p < 0.1; * p < 0.05; ** p < 0.01; *** p < 0.001.

Table 3 shows the results of Poisson regression models to estimate the associations between racial discrimination in getting testing or treatment for COVID-19 and depressive or anxiety symptoms. In unadjusted models, racial and ethnic minoritized adults who experienced racial discrimination in getting testing or treatment for COVID-19 had 1.87 times higher prevalence of depressive symptoms (95 % CI: 1.37–2.56). In the adjusted model, the magnitude of the association was similar and statistical significance remained (aPR: 1.86, 95 % CI: 1.36–2.53). However, racial discrimination in getting testing or treatment for COVID-19 was not associated with the prevalence of anxiety symptoms (PR: 1.25, 95 % CI: 0.89–1.75; aPR: 1.25, 95 % CI: 0.90–1.74).

**Table 3:** Poisson regression: Racial discrimination in getting testing or treatment for COVID-19, depressive symptoms, and anxiety symptoms among racial and ethnic minoritized adults, Michigan COVID-19 Recovery Surveillance Study, 2020–2022 (n = 1,364).

**Dependent variable:**	**Have depressive symptoms (yes/no)**		**Have anxiety symptoms (yes/no)**	
	Unadjusted PR (95 % CI)	Adjusted PR (95 % CI)	Unadjusted PR (95 % CI)	Adjusted PR (95 % CI)
Experienced racial discrimination in getting testing or treatment for COVID-19	1.87***	1.86***	1.25	1.25
	(1.37–2.56)	(1.36–2.53)	(0.89–1.75)	(0.90–1.74)
Age group (ref: ≥55)				
18–34		1.14		1.98***
		(0.79–1.63)		(1.39–2.83)
35–44		0.97		1.38
		(0.65–1.45)		(0.92–2.05)
45–54		1.25		1.48*
		(0.86–1.81)		(1.00–2.18)
Sex (ref: male)				
Female		1.00		1.41**
		(0.78–1.28)		(1.11–1.78)
Race and ethnicity (ref: Hispanic)				
Non-Hispanic Black		0.87		1.02
		(0.65–1.16)		(0.77–1.34)
Another non-Hispanic race/ethnicity		0.68*		0.90
		(0.50–0.91)		(0.69–1.19)
Marital status (ref: not married/cohabiting)				
Married/cohabiting		0.66**		0.83
		(0.51–0.85)		(0.66–1.04)
Education (ref: college graduates)				
≤High school graduates		1.42†		1.56**
		(1.00–2.01)		(1.12–2.16)
Some college		1.07		1.47*
		(0.76–1.52)		(1.08–2.00)
Household income (ref: ≥$75,000)				
<$35,000		1.00		1.32
		(0.68–1.47)		(0.92–1.89)
$35,000–74,999		1.00		1.20
		(0.68–1.47)		(0.84–1.71)
Employment status (ref: unemployed)				
Employed		1.09		1.15
		(0.84–1.41)		(0.91–1.46)
Health insurance status (ref: private insurance)				
No health insurance		1.37†		0.89
		(0.97–1.94)		(0.63–1.25)
Medicare/Medicaid/others		1.23		1.08
		(0.92–1.65)		(0.84–1.40)
Pre-existing physical comorbidities		1.31†		1.41**
		(1.00–1.72)		(1.12–1.77)
Pre-existing psychological/psychiatric condition		3.07***		2.66***
		(2.40–3.93)		(2.13–3.32)

**Notes:** COVID-19 = coronavirus disease 2019; PR = prevalence ratio; CI = confidence interval; The adjusted model included survey mode and year of COVID-19 infection

†p < 0.1; * p < 0.05; ** p < 0.01; *** p < 0.001.

## 7 Discussion

To our knowledge, the present study is the first to provide the prevalence of racial discrimination in the healthcare setting and its association with mental health outcomes in the U.S. amid the COVID-19 pandemic. Using a probability sample of adults who tested positive for SARS-CoV-2 in Michigan, we found that 7.3 % and 8.7 % of racial and ethnic minoritized adults experienced racial discrimination in seeking healthcare for COVID-19 and in getting testing or treatment for COVID-19, respectively. The prevalence is similar to a previous study prior to the COVID-19 pandemic, which reported that 5.2 % of Hispanic adults and 10.9 % of non-Hispanic Black adults experienced racial discrimination in seeking healthcare, compared to 2.0 % of non-Hispanic White adults (Hausmann et al., 2008). Although the overall prevalence of racial discrimination in Michigan healthcare settings appears to be low, we found that experiences of racial discrimination in both seeking COVID-19 healthcare and getting testing or treatment for COVID-19 were associated with depressive symptoms. This may be because racial discrimination is a social stressor that affects individuals’ social and psychological functions and behaviors, leading to depressive symptoms, as minority stress theory suggests (Harrell, 2000, Wu et al., 2021). Our result affirms previous findings of an association between racial discrimination in general settings and depressive symptoms among minoritized populations (Matthews et al., 2013, Woo and Jun, 2022). The finding also aligns with three studies before the pandemic that focused on racial discrimination in healthcare and reported associations with worse mental and physical health (Assari et al., 2017, Gee et al., 2006, Hausmann et al., 2008).

On the contrary, we did not find statistically significant associations between racial discrimination in Michigan healthcare settings and anxiety symptoms. This finding contrasts with two previous studies that showed that higher levels of racial discrimination in general settings were associated with greater anxiety symptoms among Black populations in Canada (Kogan et al., 2022) and the U.S. (Sosoo et al., 2020). This is possibly due to different measurements of racial discrimination. Both previous papers assessed racial discrimination using Black populations’ experiences of microaggressions in general settings that occurred in daily life, while this study measured racial discrimination in a specific setting, using respondents’ experiences in seeking healthcare and getting testing or treatment for COVID-19.

This research highlights a need for anti-racist discrimination policies, educational programs, and awareness efforts in healthcare settings, particularly during a pandemic. Beginning June 1, 2022, Michigan has a new requirement that healthcare providers renewing licenses or registrations must complete one hour of implicit bias training (Implicit Bias Training, 2022). The current efforts should be better embedded within educational programs by providing medical professionals with training about long-standing racism in healthcare settings and in society that has contributed to racial and ethnic health disparities (Tsai et al., 2016). Moreover, healthcare organizations should consider adding questions about experiences of racial discrimination to their patient satisfaction surveys as important indicators of healthcare quality (Bleich et al., 2021). Governments also need to promote awareness programs in the context of healthcare to address historical and contemporary structural racism, mistreatment, and health disparities. Additionally, racial and ethnic minoritized populations are less likely to obtain treatment or psychological therapy in general, even though they suffer from a higher prevalence of depression compared to White populations (World Health Organization, 2022). Intervention programs such as counseling services or community programs should prioritize access for racial and ethnic minoritized populations to minimize their stress and depressive symptoms from racial discrimination and to strengthen coping strategies and resilience-promoting factors that can reduce the risk of poor mental health.

## 8 Study limitations

This current study has several limitations. First, our sample includes individuals who received a positive PCR test for SARS-CoV-2, were recorded in the MDSS with valid contact and geographic information, and were alive when the survey sample was drawn, which may limit generalizability. As there was relatively limited access to COVID-19 PCR testing among minoritized communities (Asabor et al., 2022), especially at the beginning of the pandemic, our results may underestimate the prevalence and influence of racial discrimination in healthcare. Second, we assessed mental health outcomes using self-reported subjective measures that do not represent clinical diagnoses. Third, the sample may have recall and response bias as respondents participated in interviews between 3.4 and 5.7 months post-COVID-19 onset. The bias may be greater for respondents who completed interviews many months after disease onset. Fourth, our data is cross-sectional, so we were not able to observe causal mechanisms. Finally, we were not able to examine the differential association by race/ethnicity due to the low prevalence of the discrimination measures and small sample sizes of some of the racial and ethnic subgroups. As different racial and ethnic groups have distinct characteristics in accessing and utilizing healthcare (Kirby et al., 2006) as well as different patterns, perceptions (Sorkin et al., 2010), and contexts of discrimination (Gong et al., 2016), disaggregated analysis could have provided valuable information. Future research should prospectively study the relationship between racial discrimination in healthcare and mental health using a sample of larger and more diverse racial and ethnic groups to inform longitudinal and heterogeneous associations.

## 9 Conclusions

Our study contributes to identifying the understudied role of racial discrimination in healthcare settings on depressive and anxiety symptoms using a population-based probability sample of adults with confirmed COVID-19. Racial discrimination contributes to a disproportionately high incidence of morbidities, including mental disorders (Centers for Disease and Control and Prevention, 2023), which have the potential to worsen the effects of COVID-19, such as hospitalization, severe symptoms, and death (World Health Organization, 2022). Therefore, we should promote current efforts to reduce racial discrimination and further develop additional anti-racial discrimination policies and awareness programs in healthcare settings.

**Funding**

The Michigan COVID-19 Recovery Surveillance Study has received funding from the Michigan Department of Health and Human Services, the Michigan Public Health Institute, the University of Michigan Institute for Data Science, the University of Michigan Rogel Cancer Center, and the University of Michigan Epidemiology Department. This manuscript is supported by the Centers for Disease Control and Prevention (CDC) of the U.S. Department of Health and Human Services (HHS) funded by CDC/HHS through grant number 6 NU50CK000510-02–04 and 1 NH75OT000078-01–00.

**Disclaimer**

The contents are those of the authors and do not necessarily represent the official views of, nor an endorsement, by CDC/HHS, or the U.S. Government.

## CRediT authorship contribution statement

**Soomin Ryu:** Conceptualization, Formal analysis, Methodology, Writing – original draft. **Jana L. Hirschtick:** Conceptualization, Supervision, Writing – review & editing. **Kristi L. Allgood:** Conceptualization, Writing – review & editing. **Robert Orellana:** Conceptualization, Writing – review & editing. **Nancy L. Fleischer:** Conceptualization, Funding acquisition, Methodology, Supervision, Writing – review & editing.

## Declaration of competing interest

The authors declare that they have no known competing financial interests or personal relationships that could have appeared to influence the work reported in this paper.

## Data Availability

Although the dataset used in this study is not currently available to others, we are in the process of making a de-identified dataset and data dictionary available. Any requests for the data in the interim can be sent to the MI CReSS study team at michigan-cress@umich.edu.
